# Clinical implication of interface pressure for a new prosthetic suspension system

**DOI:** 10.1186/1475-925X-13-89

**Published:** 2014-06-30

**Authors:** Hossein Gholizadeh, Noor Azuan Abu Osman, Arezoo Eshraghi, Nasrul Anuar Abd Razak

**Affiliations:** 1 Department of Biomedical Engineering, Faculty of Engineering, University of Malaya, 50603 Kuala Lumpur, Malaysia

**Keywords:** Lower limb, Pressure, Prostheses, Transtibial, Amputation, Prosthetic liner, Prosthetic suspension, Below-knee prosthesis, Prosthetic socket, Amputees

## Abstract

**Background:**

Prosthesis suspension systems can alter the distribution of pressure within the prosthetic socket. This study evaluates a new suspension system for lower limb prostheses, and aims to compare the interface pressure and amputees’ satisfaction with the new system compared with a common prosthetic suspension system (pin/lock).

**Methods:**

Ten transtibial amputees walked at a self-selected speed on a level ground with two different suspension systems, namely the pin/lock and HOLO system. The interface pressure was measured using the F-socket transducers at the proximal, middle and distal sites of residual limb. Furthermore, subjective feedback was logged to compare two systems.

**Results:**

The pressure was significantly higher at the proximal and distal areas with the pin/lock suspension system during the swing phase of gait (*P* < 0.05). Subjective feedback also showed traction at the stump with the pin/lock system. There were no significant differences in the pressure applied to the mid-anterior and mid posterior stump for both suspension systems. However, the lateral and medial sides exhibited higher pressure with the new system during stance phase.

**Conclusions:**

The intention of this study was to deepen understanding on the effect of suspension system on the load distribution over the residual limb. The new coupling system was proved compatible with the pin/lock system in terms of suspending the leg and amputee’s satisfaction. On the other hand, the HOLO system could distribute the pressure more uniformly over the residual limb.

## Background

One of the main concerns of prosthetic rehabilitation team is non-use or limited use of prosthesis. Provision of good prosthesis based on the amputee’s functional needs and satisfaction with the device is also important [1-4].

Suspension system, including the socket, is the most important component of prosthesis, which is directly in contact with the residual limb. Unwarranted translation, rotation and piston movement between the socket and residual limb should be avoided via proper suspension [1,5-7]. Several suspension systems are available for upper and lower limb amputees. The main parts of every suspension system are 1) a soft liner and 2) a lock (coupling) system [6,8]. Most of the current suspension systems use silicone liners for suspension [1,6,9]. These silicone liners are favored by lower limb amputees as they provide a close fit to the residuum, better function, improved appearance and superior suspension [1,9]. Mostly, the silicone suspensions are attached to the rest of components (pylon, hard socket, foot, etc.) through a single distal pin, lanyard, magnetic coupling or via seal(s) that develop vacuum [7,10,11]. According to the literature, pin silicone liners apply tension distally to the residual limb and compression proximally during the swing phase of gait. The milking phenomenon is perhaps the source of the short and long-term transformations such as edema, redness, discoloration and thickening of skin, mainly at the distal end of the stump [11,12]. Pain, volume loss (atrophy) and discomfort are the consequences of this compression. Furthermore, it is difficult to use the system for the amputees with contracture or long residual limb. A vacuum or suction system (such as sleeve or Seal-In) can solve these problems. Besides, suction systems result in improved fit within the socket and reduce the quantity of pistoning within the socket in comparison to other systems [13]. Yet, ease of don and doff is a concern, particularly for the aged amputees [9,10,14]. Moreover, good manipulation skills are required to put on and off the Seal-In liner.

Even pressure distribution of is deemed ideal in a prosthetic socket. Distribution of pressure at the socket-stump interface can be influenced by suspension system and socket shape. Several studies have examined the influence of different prosthetic components and casting techniques, alignment and suspension changes on the interface pressure inside the socket [15-21]. Alignment changes had a localized effect on interface stresses [16].

It is believed that prosthetic interface pressure can determine the amputees’ comfort [11,15-20]. The load exerted on the residual limb have been evaluated either by simulation techniques [21-23] or transducers [19,24,25]. Lower limb amputees feel pressure at the socket-stump interface during activities of daily living. The soft tissue and skin of the residual limb are not adapted to load bearing; therefore, degenerative tissue ulcer might develop as a consequence of repetitive or constant pressure exerted by the socket [18]. Other skin problems may also appear such as infection, follicular hyperkeratosis, veracious hyperplasia and allergic contact dermatitis [2,26].

Two different suspension systems were compared in a study by Ali et al. [27] on transtibial amputees: the Seal-In (suction system) and the Dermo liner (pin/lock system). Less pressure was found in the socket with the Dermo liner [27]. On the other hand, the subjects experienced less problems with the Dermo liner. Therefore, it can be established that the Dermo liner provides more comfort than the Seal-In liner. Yet, the Seal-In liner offers enhanced suspension. Beil et al. [11] observed no variation in pressure difference between the suspension modes in the pin/lock and suction systems during stance phase. Nevertheless, the pin liner squeezes proximally during swing phase, while generating suction distally on the residual limb. This is the possible cause of chronic and daily skin changes with the pin users [11].

Safety, function, comfort, easy donning/doffing, durability, cosmetic appearance and cost are the key factors that should be considered in the design of prosthetic suspension. Bearing these factors in mind, a new system was developed for silicone liners using the Velcro or hook and loop [28]. The objective of current study was to compare the common system in the market (pin/lock) with the new prosthetic suspension system in terms of interface pressure (between the stump and socket) and personal feedback. It was conjectured that the new system leads to lower compression proximally and less traction at the distal residual limb compared with the pin/lock liner. It was also presumed that the new suspension system results in proper socket fit and facilitates donning and doffing.

## Methods

### Subjects

As a sample of convenience, 10 subjects were selected to participate in the study upon signing a written consent. The University of Malaya Ethics Committee issued the ethical approval. The inclusion criteria were as follows: the ability to ambulate without assistance, no ulcer on the residual limb, no volume fluctuation at the stump and use of prosthesis within the last 6 months.

### Prosthesis

**Figure 1:**
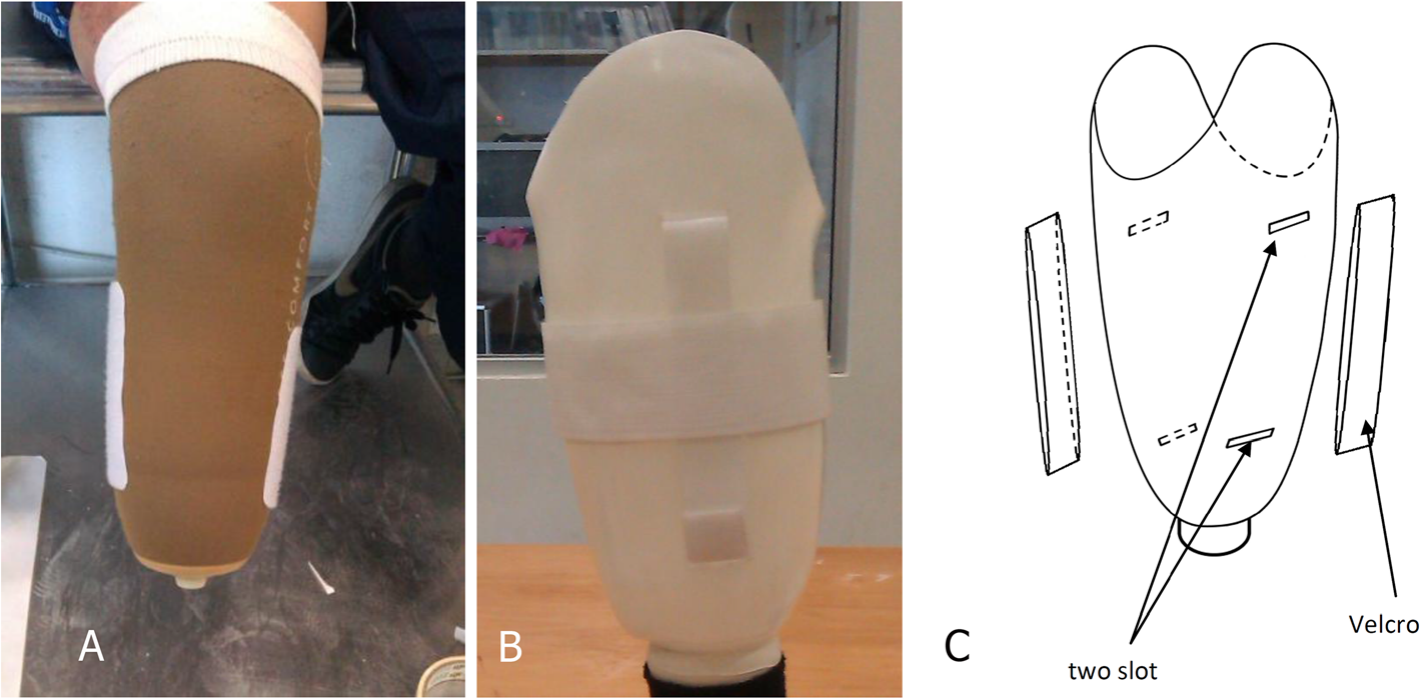
Loop attached to the silicone liner (A), and position of slots and Hook on the socket walls (B&C).

A new prosthesis with pin lock suspension system was fabricated for each participant. One of the researchers (a registered prosthetist) carried out all the processes from the casting to aligning. Flex-Foot (Talux), pylon, clamp adaptor, silicone liner and shuttle lock were used to fabricate the prostheses. A transparent check socket was manufactured to ensure total surface bearing (TSB) concept [29]. Afterwards, the subjects ambulated with the new prostheses in the laboratory (Department of Biomedical Engineering, University of Malaya, Malaysia) to become accustomed to the new foot (Flex-Foot Talux® (Össur)) and socket. Also, a 4-week trial period was given to all the participants to become fully accustomed to the new prosthesis. Afterwards, the Velcro was used as a new suspension system instead of the pin/lock mechanism (Figure 1). The pin was removed from the soft liner and the loop fastener was affixed to the silicone liner (Figure 1). The Velcro strap (hook) was attached to the socket wall (rolling part).The hook is often referred to as the male portion, while the loop is referred to as the female portion. Two small openings were created on the socket wall (medial and lateral) (Figure 1) in proximal and distal regions of the socket. We used the hook fastener (Polyester Hook & Loop Velcro V-STRONG, 100% Polyester) on the socket wall and the loop fastener on the soft liner (silicone liner) (Figure 1). This type of Velcro was chosen because it is easily accessible.

We used the same socket and alignment of the pin/lock system for the prosthesis with the new suspension. The participants were asked to use this prosthesis for 4 weeks similar to the pin/lock system to become familiar with the new suspension system. Following this trial period, the participants were required to walk on level ground with self-selected speed for the purpose of interface pressure evaluation.

### Experimental process

**Figure 2:**
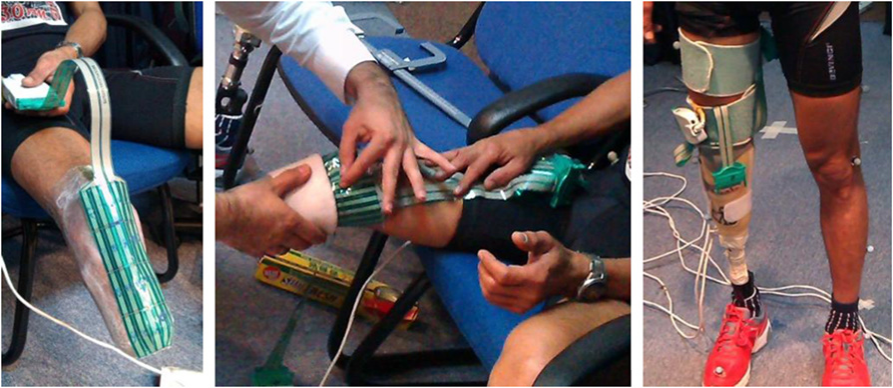
F-Socket transducer (9811E) was used in this study.

F-Socket transducers 9811E (Tekscan Inc., South Boston, USA) were used to measure the interface pressure. In general, the pressure measurement sensors for prosthesis interface should be thin. The thickness of F-socket sensors was 0.18 mm, with high resolution and good flexibility. The sensor mats were cut to match the contour of residual limb and were situated on the medial (Med), lateral (Lat), anterior (Ant) and posterior (Pos) surfaces of the stump. To prevent displacement, bonding agent (3 M Spray Mount Adhesive) was used to fix the sensors to the residuum prior to donning the silicone liners (Figure 2).

Before the experiments, the sensors were calibrated to reduce possible differences between each load cell. Equilibration and calibration were carried out according to the manufacturer’s instructions. For equilibration, the transducers were inserted separately into a bladder coupled with an air compressor and a persistent pressure was applied (100 kPa). Next, the calibration was done according to body mass. While each sensor was inside the bladder, pre and post trials were logged to ensure accurate test results. The sampling rate of pressure sensors was 50 Hz.

Force plate data was concurrently recorded to identify the gait cycle by two Kistler force plates (sampling rate of 50 Hz). The participants walked on a 10-meter walk way at a self-selected speed. Before the data collection, they practiced the experiment protocol. The participants accomplished five trials and the mean value of the middle steps was used for the analysis. The differences in peak pressure were defined within the sensor areas. Each transducer was additionally divided into proximal, middle and distal sub regions.

The individual feedback for each suspension system was also assessed in the form of a questionnaire. The first part of questionnaire evaluated the ability to don or doff the prosthesis, prosthesis fit, satisfaction during sitting, ability to walk with the prosthesis, ability to climb the stairs, and overall satisfaction [30]. The second part was related to the complaints about pistoning and rotation within the socket, sweating, pain and annoying sound. The satisfaction rate ranged from 0 to 100 (from 0 to 100, the satisfaction increased). Complaint scores of 100 indicated "not bothering" and 0 meant "extremely bothering".

### Analysis of data

For those variables that were normally distributed, we used the paired samples t test to compare pressure values. The confidence interval of 95% was set for this experiment (*P* < 0.05). SPSS software (SPSS, Chicago, IL) version 17.0 was used for the statistical analyses.

## Results

### Participants’ profile

The mean weight and age of the subjects were 76.4 kg (SD, 13.6) and 40.5 years(SD, 14.8); respectively (Table 1). The participants’ activity level was K2-K3 [31] as measured based on the American Academy of Orthotists & Prosthetists grading system. The amputation surgery for all the participants was done at least 3 years prior to the study. Table 1 presents the demographic information of participants.

**Table 1:** Subjects characteristics

**Subject no.**	**Age (Year)**	**Height (cm)**	**Mass (Kg)**	**Level of amputation**	**Cause of amputation**	**Time since amputation (year)**	**Stump length (cm)**	**Mobility grade**	**PSS**^**#**^
1	39	170	65	TT*	Traumatic	5	14	K4	Pin/Lock
2	23	167	82	TT	Traumatic	3	15	K3	Pelite
3	51	172	67	TT	Traumatic	5	14	K3	Pin/Lock
4	40	180	95	TT	Diabetic	7	16	K2	Pin/Lock
5	75	182	75	TT	Diabetic	8	13	K2	Pin/Lock
6	45	185	84	TT	Traumatic	26	12	K3	Pelite
7	41	173	95	TT	Traumatic	5	14	K3	Pin/Lock
8	34	175	78	TT	Traumatic	10	28	K3	Pin/Lock
9	32	163	72	TT	Traumatic	18	25	K2	Pin/Lock
10	25	162	51	TT	Tumour	3	16	K3	Pin/Lock

*TT = Trans-tibial.

^#^Prosthetic suspension systems used by subjects before entering to this study.

### Interface pressure

Pressure data were extracted for twelve regions of the residual limb. Table 2 presents the pressure values for the socket regions. With the pin/lock system, the proximal residuum showed slightly higher pressure (not significantly) in anterior (*P* < 0.251), posterior (*P* <0.956), and medial (*P* <0.062) regions (Table 2) during the stance phase of gait. There were no significant differences in the pressure applied to the middle of the stump for both suspension systems, except for the lateral and medial sides that exhibited significantly higher pressure with the new suspension system (*P* < 0.006 and *P* < 0.005, respectively). Furthermore, significantly higher pressure was applied to the residual limb at the distal region of the stump by the pin/lock system in anterior, posterior, and medial areas during the stance phase of gait. The pressure applied to the lateral distal stump was also higher with the pin/lock, but not significantly different (*P* < 0.092).

**Table 2:** Mean peak pressure (stance and swing) for the four major regions of the residual limb

**Descriptive statistics**								
	**Suspension type**	**N**	Mean peak pressure stance*	**Std. deviation**	**Sig**	**Mean peak pressure swing**^**#**^	**Std. deviation**	**Sig**
Anterior proximal	Pin/Lock	10	53.3	14.5	0.251	15.2	2.1	0.001*
	Holo		48.5	11.8		4.8	2.7	
Anterior middle	Pin/Lock	10	46.6	10.7	0.220	14.5	3.2	.072
	Holo		48.1	12.3		11.4	1.9	
Anterior distal	Pin/Lock	10	50.4	12.1	0.001*	24.3	2.4	0.001*
	Holo		44.5	14.2		3.1	1.1	
Posterior proximal	Pin/Lock	10	46.5	11.2	0.956	18.9	3.5	0.001*
	Holo		46.3	14.7		5.4	1.7	
Posterior middle	Pin/Lock	10	46.4	14.5	0.577	13.4	2.1	0.099
	Holo		45.8	14.1		11.2	1.8	
Posterior distal	Pin/Lock	10	62.2	19.9	0.003*	31.8	4.3	0.001*
	Holo		57.8	20.2		6.1	2.8	
Lateral proximal	Pin/Lock	10	50.1	18.9	0.434	17.3	3.1	0.001*
	Holo		51.5	19.8		7.9	2.7	
Lateral middle	Pin/Lock	10	53.9	13.5	0.006*	24.3	4.2	0.001*
	Holo		57.3	12.7		8.7	1.2	
Lateral distal	Pin/Lock	10	60.7	19.5	0.092	19.4	2.6	0.001*
	Holo		58.6	21.2		8.6	2.3	
Medial proximal	Pin/Lock	10	43.3	14.4	0.062	17.3	3.6	0.009*
	Holo		42.3	13.2		8.6	1.4	
Medial middle	Pin/Lock	10	49.3	11.9	0.005*	26.5	4.1	0.001*
	Holo		53.3	11.2		6.9	2.2	
Medial distal	Pin/Lock	10	47.8	9.6	0.003*	17.6	2.3	0.001*
	Holo		44.1	10.8		9.4	2.1	

^#^Kpa.

*= significant differences.

The results showed significantly higher pressure values at the proximal and distal residual limb using the pin/lock suspension system during the swing phase of gait. Moreover, the pressure applied to the middle stump was higher at the anterior (0.072), posterior (0.099), lateral (0.001) and medial (0.001) areas during the swing phase.

### Subjective feedback

The participants were generally satisfied with the new system (Table 3). There was no significant difference between the new system and the pin/lock system during sitting (*P* < 0.656), walking (*P* < 0.223), climbing the stairs (*P* < 0.086), and sweating (*P* < 0.586). However, the participants were content with the new system (HOLO) due to easy donning and doffing, although it was not significantly different (*P* < 0.077). Also, less movement was seen between the liner and socket. There was no traction or pain at the distal liner with new system. The HOLO created more noise compared to the pin/lock system, but not significantly higher (*P* < 0.343). The irritating noise (tearing noise from the Hook and Loop) was only heard during the doffing (Table 3).

**Table 3:** Subjective feedback with two suspension systems

	**Paired Samples Statistics**			
	**Suspension systems**	**Mean**	**Std. Deviation**	**Sig. (2-tailed)**
Satisfaction				
Fit	Pin/Lock	77.5	3.0	.012*
	Holo	81.9	3.2	
Donning/Doffing	Pin/Lock	75.3	4.6	.077
	Holo	76.7	4.9	
Sitting	Pin/Lock	79.1	5.1	.656
	Holo	79.8	3.1	
Walking	Pin/Lock	76.0	2.9	.223
	Holo	76.8	2.7	
Stair	Pin/lock	75.8	3.0	.086
	Holo	77.7	1.9	
Problem				
Sweating	Pin/Lock	73.3	3.5	.586
	Holo	72.7	4.2	
Pistoning	Pin/Lock	79.3	3.8	.020*
	Holo	84.1	4.6	
Rotation	Pin/Lock	80.1	2.5	.002*
	Holo	83.5	3.2	
Sound	Pin/Lock	72.7	3.1	.343
	Holo	70.3	2.7	
Pain	Pin/Lock	77.0	2.7	.062
	Holo	79.4	3.9	
Overall satisfaction				
	Pin/Lock	76.3	1.1	.015*
	Holo	78.7	3.4	

Note: The satisfaction rate ranged from 0 to 100 (from 0 to 100, the satisfaction increased). Complaint scores of 100 indicated "not bothering" and 0 meant "extremely bothering".

*= significant differences.

## Discussion

Proper prosthetic rehabilitation relies on understanding the biomechanics of pressure between the socket and residual limb among other factors. Appropriate fit and suspension of the socket for individuals with lower limb amputation have substantial roles in the rehabilitation [32]. The clinicians need to be conscious about the effects of various suspension methods and prosthetic socket designs on residual limb and user satisfaction. The interface pressure of various prosthetic sockets has been evaluated [15,24,33-36]. The level of user satisfaction with a prosthesis is very much reliant on the appropriate pressure at the pressure-tolerant and pressure-relief areas of the residuum. This research evaluated the effect of a new suspension system (HOLO) on the pressure distribution inside the socket compared with the pin/lock suspension system.

In both systems, the pressure distribution was almost even at the anterior, posterior, medial and lateral surfaces during the stance (Table 2). Less than 100 kPa average peak pressure was seen during the gait cycle. This reflected the outcomes of preceding studies on the TSB systems [11,20,37]. Pressure at the distal area of residual limb was higher than the proximal area (not the anterior side) throughout the stance with both systems. This is consistent with the findings of Dumbleton et al. [20].

Prosthesis is suspended through application of pressure at various sites of stump. This can considerably affect the comfort during ambulation. The pin/lock users experience traction at the distal stump during the swing phase [11]. Simultaneously, proximal tissues bear high compression that may interrupt the fluid stream. This phenomenon may cause vein problems and edema. It can also result in the color change and skin thickening, especially at the distal area of the residual limb [11]. This study conjectured that increased contact area with the HOLO system may decrease the stretch. Significant differences were observed at different stump surfaces (Table 2). Less peak pressures were seen at the proximal and distal residual limb on all surfaces with the HOLO system during the swing phase of gait. This was compatible with the results of Beil and Street [11] Beil and Street [11] reported more uniform interface pressure with a suction system [11]. The current research is in line with their findings as the distribution of pressure with the pin/lock was less uniform in comparison to the HOLO system; yet HOLO is not a suction system. Similar to the suction system, the residual limb had higher contact with the socket in the new system compared with the pin/lock suspension. High contact between the socket and stump could produce more uniform pressure. In HOLO, the pressure was mostly concentrated at the middle of the residual limb; similar to the Seal-In liner [27]. This might be due to the location of the Velcro in the new system compared with the seal area in the Seal-In system. This was compatible with the findings of Ali et al. [27].

According to the literature, the Seal-In suspension system causes minimum pistoning inside the socket in comparison to the pin/lock suspension [32,36]. Additionally, subjective feedback showed that less piston movement was created by the new suspension system within the socket. This study revealed higher magnitudes of pressure with the HOLO similar to the Seal-In liner at the middle stump [27].

The PEQ is widely used to assess satisfaction with prosthesis and it has good reported validity and reliability [30]. We used only some items of this questionnaire in this study. The soft silicone liner is attached to the socket only by a distal pin in the pin/lock systems; therefore, the users feel pain and distal end traction, primarily during the swing phase of gait [12,32]. Socket fit was stated to be lower compared to the new system. Yet, the users were generally satisfied with the new system owing to the easy procedure of donning and doffing (Table 3).The prosthesis use can change tremendously depending on the ease of donning and doffing, particularly in relation to the night-time toilet habits [9,28,30,32]. Firm bound between the socket walls and soft liner in the Seal-In liners may produce a sense of confidence for the users during walking [32]. However, donning and doffing is a demanding task, mainly for the elderly or amputees with upper limb disorders, such as stroke. In the new system, the liner is fixed firmly to the socket walls like the Seal-In liners; yet, the donning and doffing is as easy as with the pin/lock system [28]. Based on the literature, it can be difficult for amputees with long residuum to use the pin/lock system (transfemoral, transtibial and knee disarticulation). Similarly, if the user has stump contracture, it can be challenging to align the pin. With the HOLO [28], extra space is not needed at the end of socket and it is a good option for residual limbs with long length and contracture.

Lanyard suspension system (US 20050256589 A1) comprises a lanyard cord that is attached to the distal part of the silicone liner, similar to the pin/lock system. Also, a lanyard lock mechanism is attached to the end of the prosthetic socket. In this system, the silicone liner is fixed inside the socket by only a distal cord and the liner can easily rotate inside the socket or crate milking similar to the pin/lock system. But, in the Holo system, two Velcros (medial and lateral sides of the liner) fix the liner inside the socket and the liner is in contact with the socket on most of its surface. This could eliminate the rotation and milking problems.

### Limitation and strength

Variation in residual limb dimensions may affect the pressure distribution; thus, a larger sample size is needed to find possible relationships between the dimension of residual limb and pressure distribution. The pressure profile can be also compared for various activities and walking surfaces.

In this study, a registered prosthetist carried out all the processes from the casting to aligning the new prostheses. We used same socket, prosthetic components (foot, pylon, and silicone liner) and alignment for both suspension systems to decrease the bias in our results.

## Conclusions

This study attempted to provide a vision on pressure alteration with different prosthetic suspension systems. The HOLO system may distribute the pressure more uniformly compared with the pin/lock system, especially during the swing phase of gait.

## Abbreviations

Ant: Anterior; HOLO: Hook and loop; kg: Kilogram; kPa: Kilo Pascal; Lat: Lateral; Med: Medial; Mm: Millimeter; PEQ: Prosthesis evaluation questionnaire; Pos: Posterior; SD: Standard deviation; TSB: Total surface bearing; TT: Trans-tibial.

## Competing interests

The authors declare no conflict of interests.

## Authors’ contributions

H.G designed the system and the protocol, fabricated the prostheses, conducted the experiments, collected and analyzed the data, discussed the results and drafted the manuscript. N.A.A.O supervised the overall project, and helped in revising the manuscript. A.E and N.A.A.R collected and analyzed the data, discussed the results, prepared some parts of the manuscript. All authors read and approved the final manuscript.
